# Effects of fermented rice husk powder on growth performance, rumen fermentation, and rumen microbial communities in fattening Hu sheep

**DOI:** 10.3389/fvets.2024.1503172

**Published:** 2024-11-27

**Authors:** Yanming Cheng, Han Zhang, Jiawei Zhang, Hongwei Duan, Yuyang Yin, Yufeng Li, Shengyong Mao

**Affiliations:** ^1^Laboratory of Gastrointestinal Microbiology, National Center for International Research on Animal Gut Nutrition, Nanjing Agricultural University, Nanjing, China; ^2^Ruminant Nutrition and Feed Engineering Technology Research Center, College of Animal Science and Technology, Nanjing Agricultural University, Nanjing, China; ^3^Huzhou Academy of Agricultural Sciences, Huzhou, China

**Keywords:** fermented rice husk, Hu sheep, growth performance, rumen fermentation, rumen microbiota

## Abstract

**Introduction:**

This study aimed to examine the effects of fermented rice husk powder feed on growth performance, apparent nutrient digestibility, and rumen microbial communities in fattening Hu sheep.

**Methods:**

Twenty-one male Hu sheep with similar body weights (32.68 ± 1.59 kg) were randomly assigned to three groups: a control group (CON) receiving a TMR with soybean straw, a rice husk powder group (RH), and a fermented rice husk powder group (FHR).

**Results:**

The results indicated that the FHR group exhibited a significant increase in ADG and FBW of Hu sheep compared to the other two groups (*p* < 0.05). The digestibility of CP and EE was significantly higher in the CON and FHR groups than in the RH group (*p* < 0.01). Furthermore, the digestibility of DM in the CON group was higher than in the FHR and RH groups (*p* < 0.01). The FHR group showed lower NDF and ADF digestibility compared to the CON group, but higher than the RH group (*p* < 0.05). Additionally, serum ALB and ALT levels in the CON group were elevated compared to those in the two groups (*p* < 0.05). The rumen concentrations of TVFA, butyrate, and valerate in the FHR group were significantly elevated compared to the other two groups (*p* < 0.05). At the genus level, the relative abundances of *Rikenellaceae RC9 gut group*, *Succinimonas*, *UCG-010_norank*, *UCG-005*, *p-251-o5_norank*, and *Lachnospiraceae AC2044 group* were significantly diminished in the FHR group compared to the CON group (*p* < 0.05). In contrast, the relative abundance of *Succinivibrio* was significantly higher (*p* < 0.05), while the abundances of *Eubacterium coprostanoligenes group_norank* and *Quinella* were significantly lower (*p* < 0.05) in the RH group compared to the CON group. Spearman correlation analysis revealed negative correlations between the *Rikenellaceae RC9 gut group* and propionate, butyrate, and TVFA, as well as between *Prevotellaceae UCG-003* and both propionate and TVFA. Conversely, *Ruminococcus* showed a positive correlation with propionate and TVFA.

**Discussion:**

In conclusion, replacing 15% of soybean straw with fermented rice husk powder feed modified the rumen microbiota and improved the growth performance of fattening Hu sheep.

## 1 Introduction

Roughage is an essential feed ingredient for ruminants, not only providing substantial nutrients but also playing a critical role in maintaining rumen health (1). In China, due to the scarcity of high-quality forages, large quantities of forage such as alfalfa and oat grass are imported annually to meet ruminant production needs (2). However, while high-quality roughage is limited, a large amounts of agricultural by-products, such as straw and rice husks, remain underutilized (3). Rice husk, a major by-product of rice milling, is abundant and inexpensive, with an annual production of approximately 40 million tons in China (4). Research indicates that the cellulose content in rice husks is high and has potential to partially replace traditional roughage for ruminants (5). However, the coarse and hard texture of rice husks results in poor palatability, low intake, and low digestibility when fed directly (6). Current methods for processing agricultural by-products, including physical and chemical approaches, have not been widely adopted due to high energy consumption, environmental pollution, and immature technology (7). How to use rice husk powder to fill the gap in roughage resources has become a new challenge.

In recent years, biological fermentation methods have shown promising results in reducing the fiber content of agricultural by-products and enhancing their feed value (8). Microbial fermentation, a common method for improving feed palatability and nutritional value, works by decomposing macromolecules into a range of metabolites that promote digestion and health while reducing harmful substances in the feed (9). Previous studies have demonstrated that microbial fermentation can improve feed palatability and significantly increase nutrient content, leading to improved growth indicators in animals (10, 11). For instance, adding fermented by-products to lamb starter feeds has been shown to increase average daily gain and enhance the richness and diversity of rumen microbial communities (12). Similarly, incorporating fermented mushroom residues into total mixed rations (TMR) has significantly improved production performance, meat quality, and the diversity and abundance of rumen bacterial communities in Hu sheep (13). Our initial experiments determined the optimal strains and fermentation conditions for probiotic fermentation of rice husk powder (14). Post-fermentation, a significant reduction the pH of the rice husk powder was observed, alongside a notable increase in the viable microbial count. Additionally, the contents of acid-soluble protein and crude fat exhibited substantial increases of 118.05 and 60.44%, respectively (14). Given the observed enhancements in fermentation indices and nutritional content, we hypothesized that compound fermented rice husk powder may positively impact the growth of meat sheep. Therefore, this study aimed to evaluate the effects of partially substituting soybean straw with fermented rice husk powder on sheep growth performance, nutrient digestibility, and rumen microbiota. The findings were intended to offer theoretical guidance for the safe and efficient utilization of fermented rice husk powder.

## 2 Materials and methods

### 2.1 Animals, diets, and experimental design

The experimental protocol was approved by the Animal Care and Use Committee of Nanjing Agricultural University (protocol number: SYXK2017-0007).

A total of 21 healthy male Hu sheep in the fattening stage with an average body weight (BW) of 34.19 ± 1.40 kg were selected for the study and randomly allocated into three groups, each consisting of seven sheep. The experimental groups included a control group (CON), a rice husk powder group (RH), and a fermented rice husk powder group (FHR). The control group was fed a total mixed ration (TMR) containing soybean straw powder, while the rice husk powder group replaced 15% of the soybean straw powder in the TMR with rice husk powder feed (composed of 80% rice husk powder, 15% corn grain, and 5% soybean meal). The fermented rice husk powder group substituted 15% of the soybean straw powder in the TMR with fermented rice husk powder feed. The diets were formulated according to nutrient requirements of meat-type sheep and goat (NY/T 816–2021) (15). All raw materials were procured from Da Bei Nong Technology Co., Ltd. in Anhui Province. Ingredients and chemical composition of the experimental diets are presented in Table 1. Each sheep was housed in an individual pen (1.5 m x 2 m) with wooden slatted floors and had free access to drinking water. All the sheep were fed twice daily at 07:00 and 16:00, ensuring a surplus of 5–10%. The pre-feeding period lasted for 7 days, followed by a formal experimental period of 35 days. All sheep were uniformly dewormed prior to the experiment.

**Table 1:** Ingredients and chemical composition of the experimental diets.

Items	Groups		
	CON	RH	FHR
Ingredients, % DM			
Corn grain	26.00	28.25	26.00
Cottonseed meal	4.00	4.00	4.00
Soybean meal	3.00	3.75	3.00
Malt sprouts	5.00	5.00	5.00
Wheat bran	7.00	7.00	7.00
Corn bran	10.00	10.00	10.00
Soybean hull	10.00	10.00	10.00
Soybean straw powder	25.50	10.50	10.50
Rice husk powder	—	12.00	—
Fermented rice husk powder feed^1^	—	—	15.00
Calcium carbonate	0.80	0.80	0.80
Salt	0.80	0.80	0.80
Calcium monophosphate	0.80	0.80	0.80
Distillers’ Grains	5.00	5.00	5.00
Premix^2^	2.10	2.10	2.10
Nutrient composition, % DM			
CP	16.32	16.45	16.83
EE	4.77	4.40	4.57
NDF	48.30	45.25	44.60
ADF	26.00	25.59	26.00
ME, MJ/kg DM	9.32	9.40	9.30
Ca	0.76	0.79	0.78
P	0.60	0.67	0.64

^1^The fermented rice husk powder feed consists of a fermented substrate consisting of 80% rice husk meal, 15% corn grain and 5% soybean meal. ^2^Formulated to provide (per kilogram of premix): 150000KIU of vitamin A, 100000 KIU of vitamin D_3_, 600 IU of vitamin E, 0.3 g of Cu, 1.2 g of Fe, 1.8 g of Mn, 25 mg of I, 10 mg of Se, 15 mg of Co.

The probiotics incorporated in the fermented rice husk powder feed, such as *Lactobacillus plantarum* L1, *Bacillus subtilis* B6, and *Saccharomyces cerevisiae* Y2, were sourced from Nanjing Zhirun Biotechnology Group Co., Ltd. The inoculation process employed a ratio of 1:2:2 for *Lactobacillus plantarum* L1, *Bacillus subtilis* B6, and *Saccharomyces cerevisiae* Y2, respectively, with the probiotic solution constituting 7% of the total feed volume. To facilitate optimal fermentation, the moisture content of the feed was maintained at 35%. The fermentation process occurred under controlled conditions at 25°C for 96 h, following the method outlined by Cheng et al. (14). The nutritional compositions of both the rice husk powder feed and the fermented rice husk powder feed are provided in Appendix 1.

### 2.2 Sampling and measurement

#### 2.2.1 Growth performance

On the first day prior to the formal experimental period and on the last day of the experimental period, the sheep were weighed before the morning feeding to calculate the average daily gain (ADG). Additionally, the diet offered and the orts were measured daily for each group of sheep to assess dry matter intake (DMI) and the feed-to-gain ratio (F/G) of the Hu sheep.

#### 2.2.2 Apparent nutrient digestibility

During the digestibility assessment, each sheep was individually housed in a pen. On the last day of each week of the formal experimental period, fecal samples were collected directly from the rectum. Collections occurred twice daily, once in the morning and once in the evening. The samples were thoroughly homogenized, a portion was combined with an equal volume of 10% dilute sulfuric acid for nitrogen fixation. All fecal samples were stored at −20°C.

During the concluding 3 days of the experiment, feed samples were collected, thoroughly mixed, and subjected to the quartering method for sampling. After the experiment, both fecal and feed samples were dried in an oven at 65°C for 48 h. Subsequently, the samples were ground using a Cyclotec mill (Tecator 1,093; Tecator AB, Höganäs, Sweden) with a 40-mesh sieve for conventional nutrient analysis. A portion of the air-dried feed was further dried at 105°C for 3 h to determine the dry matter content. The methods used to measure neutral detergent fiber (NDF) and acid detergent fiber (ADF) were based on Van Soest et al. (16), while the crude protein (CP), crude fat (EE), and ash content in feed and feces were measured according to AOAC (17). The apparent digestibility was calculated using acid-insoluble ash (AIA) as a marker, following the methods of Van Keulen and Chaney (1977). The calculation formula is as follows:


$$\mathrm{Nutrient digestibility}\;\left(\%\right)=\left[\begin{array}{l} 1-\left(\begin{array}{l} AIA\;\mathrm{concentration in feed}/\\ AIA\;\mathrm{concentration in feces} \end{array}\right)\times \\ \left(\begin{array}{l} \mathrm{Nutrient concentration in}\\ \;\mathrm{feces}/\mathrm{Nutrient concentration in feed} \end{array}\right) \end{array}\right]\times 100\%$$

#### 2.2.3 Serum biochemical indices

On the 34th day of the formal experimental period, blood samples were collected from the jugular vein using a vacuum tube 2.5 h post-morning feeding. Following clotting, the blood samples were centrifuged at 3500 × g for 15 min at 4°C, and the serum was stored in liquid nitrogen for subsequent analysis of aspartate aminotransferase (AST), alanine aminotransferase (ALT), and alkaline phosphatase (ALP), total protein (TP), albumin (ALB), Urea, glucose (GLU), total cholesterol (TCHO), and triglycerides (TG). Serum biochemical indices were measured using the Beckman Coulter AU5800 automatic biochemical analyzer (United States). The globulin concentration (GLB) was calculated by subtracting the albumin concentration from the total protein concentration.

#### 2.2.4 Rumen fermentation parameters

On the 35^th^ day of the formal experimental period, rumen fluid was collected 2.5 h post-morning feeding using an oral stomach tube. The first 50 mL of the sample was discarded to minimize contamination from saliva. Following immediate filtration through four layers of cheesecloth, the pH of the rumen fluid was measured using a portable pH meter (HI-9024C, HANNA Instruments, United States). The remaining samples were subpackaged and stored at −20°C for subsequent analysis. 1 mL of rumen fluid was mixed with 0.2 mL of 25% (w/v) orthophosphoric acid and analyzed for VFA concentrations using gas chromatography (GC-14B, Shimadzu, Japan) (18). The ammonia nitrogen (NH_3_-N) concentration in the rumen fluid was measured using a colorimetric method (19). Microbial crude protein (MCP) concentration was determined using a Bradford protein concentration assay kit (Beijing Solarbio Science & Technology Co., Ltd).

#### 2.2.5 DNA extraction, 16S rRNA amplicon sequencing, and bacterial composition analysis

Total microbial genomic DNA was extracted from rumen content samples using the E.Z.N.A.® soil DNA Kit (Omega Bio-Tek, Norcross, GA, United States) according to the manufacturer’s instructions. The quality and concentration of DNA were determined by 1.0% agarose gel electrophoresis and a NanoDrop® ND-2000 spectrophotometer (Thermo Scientific Inc., United States) and kept at −80°C prior to further use. The hypervariable region V3-V4 of the bacterial 16S rRNA gene was amplified with primer pairs 338F: ACTCCTACGGGAGGCAGCAG and 806R: GGACTACHVGGGTWTCTAAT (20) by an ABI Gene Amp® 9,700 PCR thermocycler (ABI, CA, United States). Purified amplicons were pooled in equimolar amounts and paired-end sequenced on an Illumina Mi Seq PE300 platform/Nova Seq PE250 platform (Illumina, San Diego, USA) according to the standard protocols by Majorbio Bio-Pharm Technology Co. Ltd. (Shanghai, China).

Raw FASTQ files were de-multiplexed using an in-house Perl script, then quality-filtered by fastp version 0.19.6 (21) and merged by FLASH version 1.2.7 (22) with the following criteria: The 300 bp reads were truncated at any site receiving an average quality score of <20 over a 50 bp sliding window, and the truncated reads shorter than 50 bp were discarded. Reads containing ambiguous characters were also discarded. Only overlapping sequences longer than 10 bp were assembled according to their overlapped sequence. The maximum mismatch ratio of the overlap region is 0.2. Reads that could not be assembled were discarded. Then the optimized sequences were clustered into operational taxonomic units (OTUs) using UPARSE 7.1 (23) with a 97% sequence similarity level. The most abundant sequence for each OTU was selected as a representative sequence. On the basis of the above analyses, a series of in-depth statistical and visual analyses, such as multivariate analysis and difference significance test, were conducted on the community composition of multiple samples. Alpha diversity was calculated using Qiime software (Version 1.9.1), and differences between groups were analyzed using R software (Version 2.15.3). Bray-Curtis distances were computed using the default script from the Phyloseq package to measure beta diversity. Principal Component Analysis (PCoA) was conducted using the ade4 and ggplot2 packages of R software.

### 2.3 Statistical analyses

The growth performance, apparent digestibility, rumen fermentation parameters, and serum biochemical indicators of Hu sheep were analyzed using one-way ANOVA in SPSS 26.0, and multiple comparison tests (SNK method) were performed. Covariance analysis was used for the FBW, with the covariate being the IBW. The differences in alpha diversity indicators and relative abundance of microbial communities were analyzed using non parametric tests (Kruskal Wallis). Spearman’s rank correlation coefficients were calculated between the relative abundances of all pairs of genera using the Hmisc package in R (version 4.0.3). Only significant correlations (*p <* 0.05) with a Spearman’s correlation coefficient |R| > 0.6 were retained for further analysis. An adjacency matrix based on significant Spearman correlations was created to represent the network. The network was visualized using Gephi. Nodes in the network represented genera, and edges represented significant correlations between them. Node size was proportional to the genus’s relative abundance, and edge thickness was proportional to the strength of the correlation. The significance level *p <* 0.05 indicates significant differences, while *p <* 0.01 indicates extremely significant differences in the data.

## 3 Results

### 3.1 Growth performance

No significant differences in IBW, DMI, and F/G were observed among the three groups of Hu sheep (*p >* 0.05). However, there was a significant improvement in FBW (*p <* 0.05). Compared to the RH group, the ADG in the FHR group was significantly higher (*p <* 0.05), while no significant differences were noted between the FHR and control groups (*p >* 0.05) (Table 2).

**Table 2:** Effects of fermented rice husk powder on growth performance in fattening Hu sheep.

Items	Groups			SEM	*P*-value
	CON	RH	FHR		
DMI, g/d	1453.1	1438.6	1608.2	35.03	0.084
IBW, kg	32.63	32.69	32.71	0.36	0.996
FBW, kg	38.54^b^	37.86^b^	39.76^a^	0.40	0.039
ADG, g/d	166.5^ab^	147.8^b^	204.5^a^	9.70	0.043
F/G	9.04	9.97	8.21	0.38	0.166

CON (Control diet), RH (Containing 15% rice husk powder feed), FHR (Containing 15% fermented rice husk powder feed).

### 3.2 Apparent digestibility of nutrients

The apparent digestibility of CP and EE was significantly higher in the FHR and control groups compared to the RH group (*p <* 0.001), with no significant difference observed between the FHR and CON groups (*p >* 0.05). The apparent digestibility of DM, NDF, and ADF was significantly lower in the RH and FHR groups compared to the CON (*p <* 0.001). No significant difference in DM digestibility was found between the RH and FHR groups. However, the digestibility NDF and ADF was significantly higher in the FHR group compared to the RH group (*p <* 0.05) (Table 3).

**Table 3:** Effects of fermented rice husk powder on nutrient apparent digestibility in fattening Hu sheep.

Items	Groups			SEM	*P*-value
	CON	RH	FHR		
DM, %	62.40^a^	58.86^b^	59.64^b^	0.54	<0.001
CP, %	75.28^a^	69.87^b^	75.42^a^	0.59	<0.001
EE, %	67.36^a^	60.42^b^	68.69^a^	1.21	<0.001
NDF, %	47.23^a^	29.49^c^	34.47^b^	1.34	<0.001
ADF, %	33.76^a^	16.47^c^	22.80^b^	1.41	<0.001

CON (Control diet), RH (Containing 15% rice husk powder feed), FHR (Containing 15% fermented rice husk powder feed).

### 3.3 Serum biochemical indices

In comparison to the CON group, the levels of ALB and ALT were significantly decreased in both the RH and FHR groups (*p <* 0.05). However, no significant differences were observed among the three groups for other indices, including AST, TP, GLOB, ALP, Urea, GLU, TCHO, and TG (*p >* 0.05) (Table 4).

**Table 4:** Effects of fermented rice husk powder on serum biochemical parameters in fattening Hu sheep.

Items	Groups			SEM	*p*- value
	CON	RH	FHR		
TP, g/L	60.33	56.26	56.55	1.03	0.203
ALB, g/L	27.11^a^	24.11^b^	24.87^b^	0.53	0.046
GLB, g/L	33.21	32.14	31.69	0.81	0.750
A/G	0.83	0.79	0.80	0.02	0.697
UREA, mmol/L	9.08	8.47	9.84	0.31	0.209
TCHO, mmol/L	1.39	1.38	1.51	0.05	0.550
TG, mmol/L	0.30	0.37	0.42	0.03	0.244
GLU, mmol/L	4.43	5.64	5.53	0.29	0.168
ALP, U/L	546.7	415.6	457.4	26.49	0.116
ALT, U/L	19.71^a^	15.14^b^	15.71^b^	0.81	0.034
AST, U/L	102.7	89.86	100.00	3.29	0.253

CON (Control diet), RH (Containing 15% rice husk powder feed), FHR (Containing 15% fermented rice husk powder feed).

### 3.4 Rumen fermentation parameters

**Figure 1:**
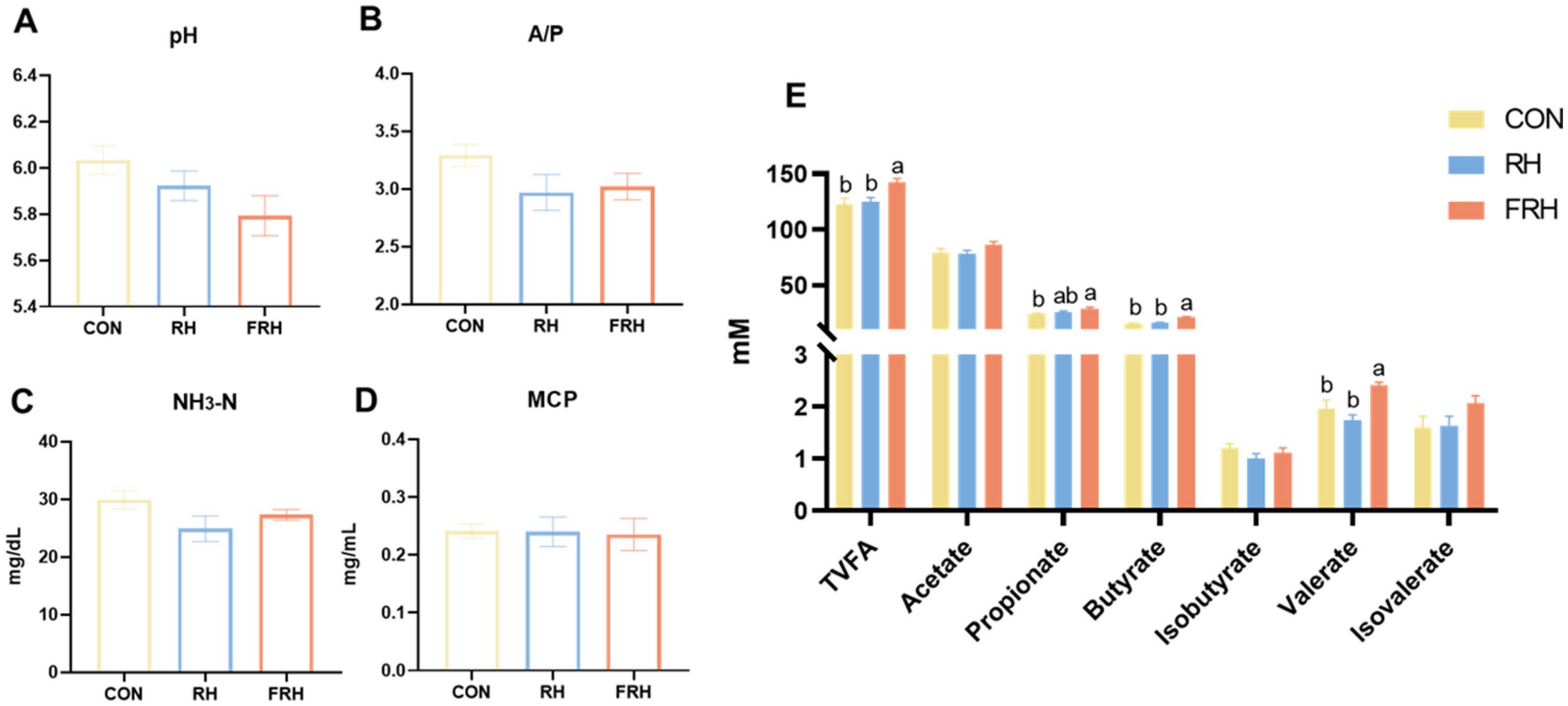
Effects of fermented rice husk powder on rumen fermentation parameters in fattening Hu sheep. (A) rumen pH, (B) A/P = acetate to propionate ratio, (C) NH3-N = ammonia nitrogen, (D) MCP = microbial protein, (E) rumen VFA.

There were no significant differences in rumen pH among the three groups (*p >* 0.05), but the FHR group had the lowest pH. Concentrations of TVFA, butyrate, and valerate were significantly elevated in the FHR group compared to the other two groups (*p <* 0.05). Both the RH and FHR groups exhibited a significant decrease in the A/P compared to the CON group (*p <* 0.05). No significant differences were observed in the concentrations of acetate, propionate, NH_3_-N, and MCP among the three groups (*p >* 0.05; Figure 1).

### 3.5 Rumen microbial diversity

**Figure 2:**
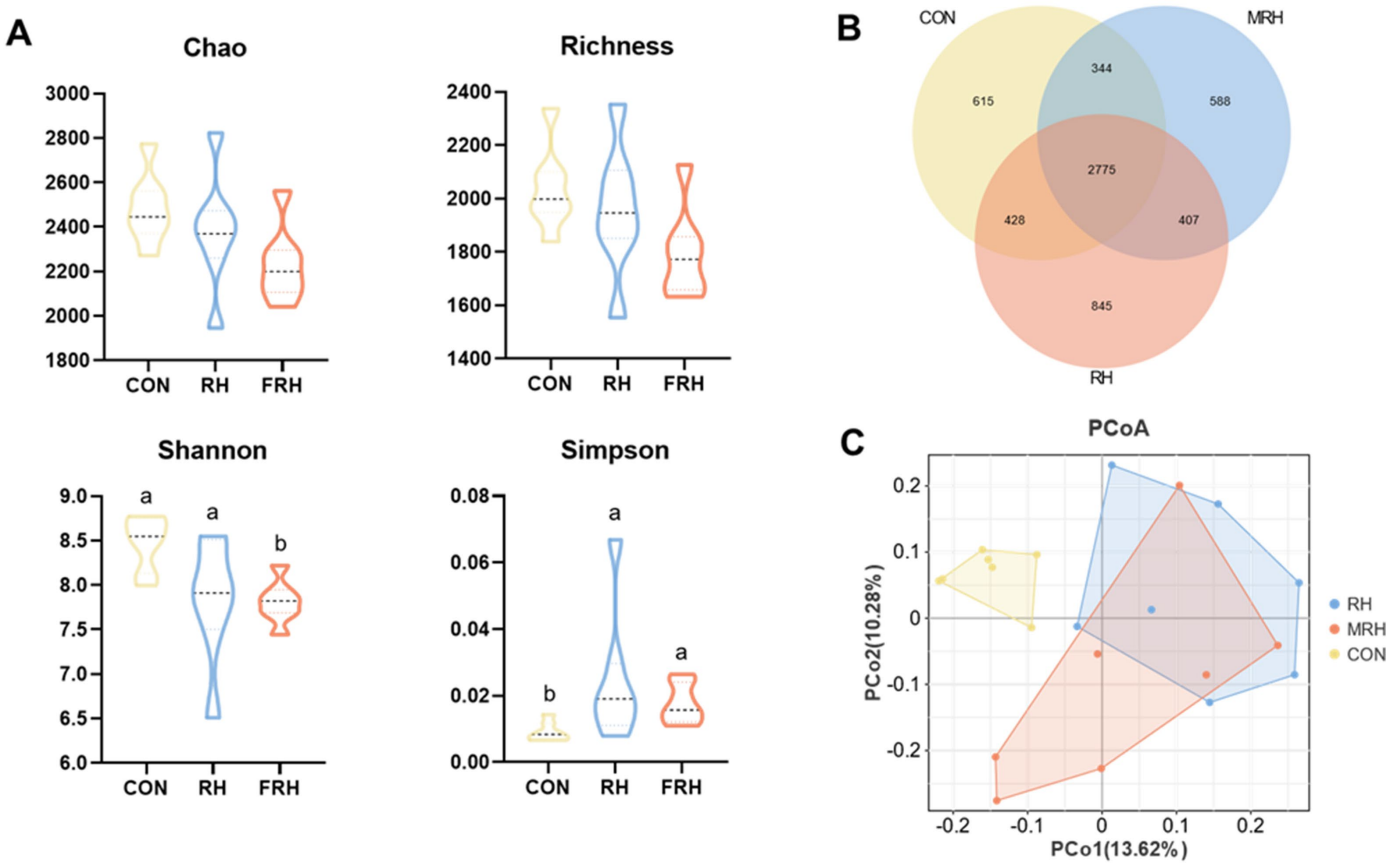
Effects of fermented rice husk powder on rumen microbial diversity in fattening Hu sheep. (A) Alpha diversity analysis, (B) Venn diagram and microbial operational taxonomic unit (ASV), (C) Bray-Curtis PCoA by rumen bacteria.

In terms of alpha diversity, no significant differences were observed in the ACE and Chao1 indices among the CON, RH, and FHR groups (*p >* 0.05). However, the FRH and RH groups significantly increased the Simpson index (*p <* 0.05), while the Shannon index of the FHR group was lower (*p <* 0.05). The PCoA results based on the Unweighted-UniFrac metric demonstrated a distinct separation of the FHR group from both the CON and RH groups (R = 0.388, *p* = 0.001) (Figure 2).

### 3.6 Rumen fermentation parameters

**Figure 3:**
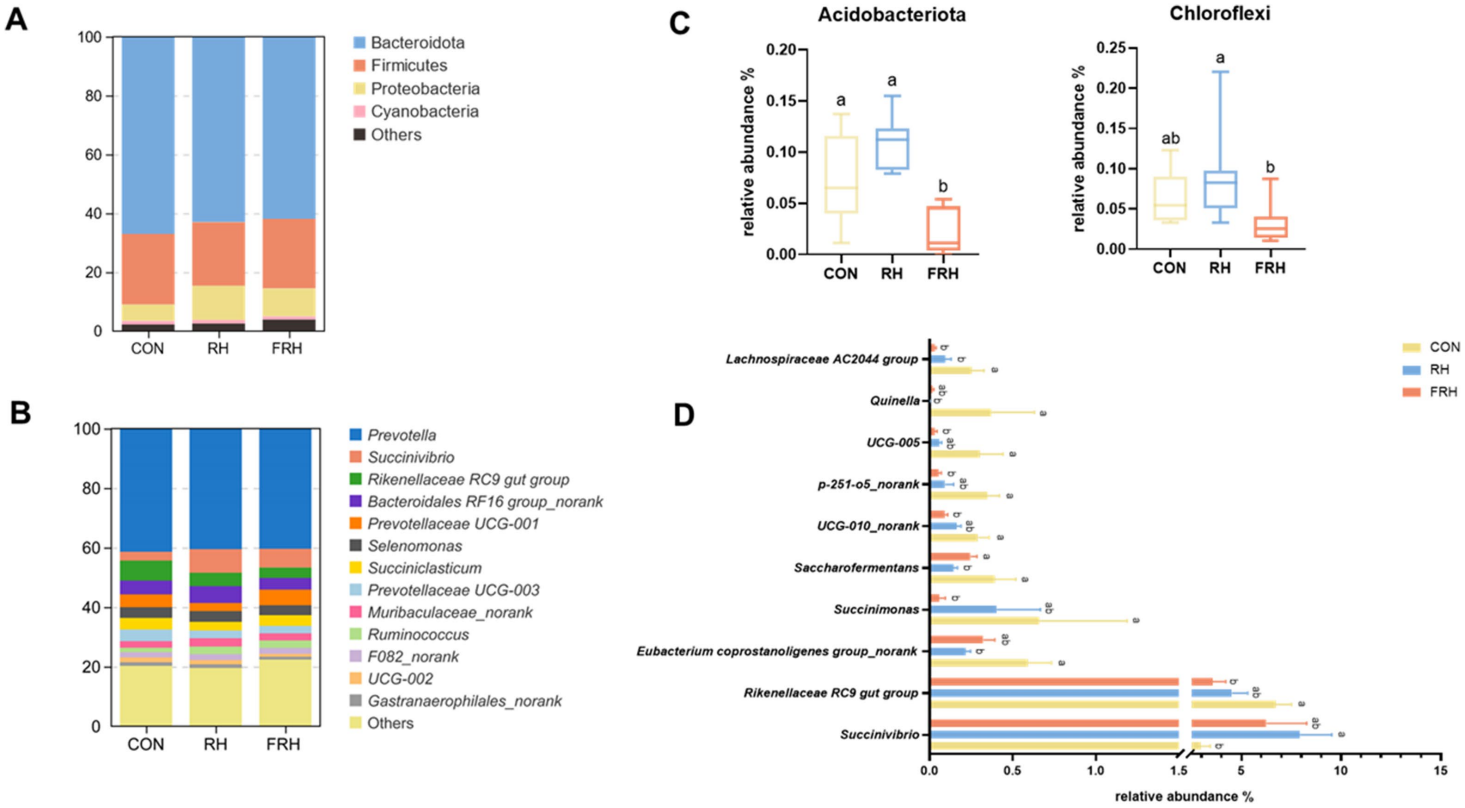
Effect of fermented rice husk powder on the relative abundance (relative abundance >1% in at least one treatment) of rumen bacteria at phylum (A) and genus (C) levels in fattening Hu sheep. (B) Differential rumen bacteria at the phylum level. (D) Differential rumen bacteria at the genus level.

A total of 29 phyla were identified, with Bacteroidota (63.97%), Firmicutes (23.10%), Proteobacteria (8.87%), and Cyanobacteria (1.15%) having a relative abundance greater than 1% (Figure 3A). The relative abundances of Acidobacteriota and Chloroflexi in the FHR group were lower than in the RH group, although no differences were noted when compared with the CON group (Figure 3B).

At the genus level, 13 genera had a relative abundance greater than 1%. The FHR group exhibited a significantly lower relative abundance of *Rikenellaceae RC9 gut group*, *Succinimonas*, *UCG-010_norank*, *UCG-005*, *p-251-o5_norank*, and *Lachnospiraceae AC2044 group* compared to the CON group (*p <* 0.05). Conversely, the relative abundance of *Succinivibrio* in the RH group was significantly higher than that in the CON group (*p <* 0.05), while the relative abundances of *Eubacterium coprostanoligenes group_norank* and *Quinella* were lower than in the CON group (*p <* 0.05). However, no differences were found in the relative abundances of these genera between the FHR and RH groups (*p >* 0.05; Figures 3C,D).

### 3.7 Relationships between major rumen bacteria, fermentation parameters, and growth performance

**Figure 4:**
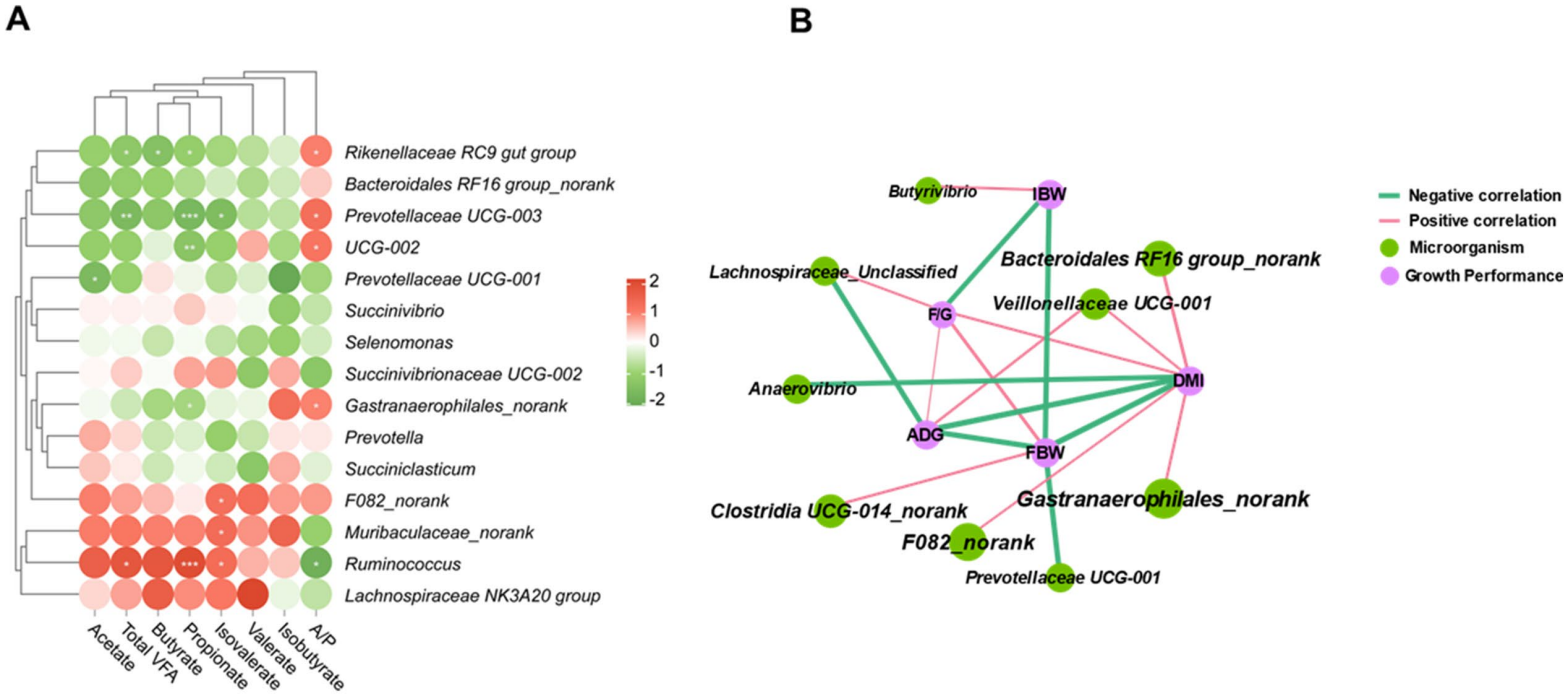
Relationships of bacterial communities, VFA fermentation parameters and growth performance. The heatmap shows that the correlation between the predominant rumen bacteria (relative abundance of the top 15) and VFA fermentation parameters (A), the networks are constructed based on the adjacency matrix of microbial abundance and growth performance from the correlation analysis (B) (*p* < 0.05, R > 0.6).

Spearman correlation analysis was conducted to investigate the relationships between rumen VFA and the top 15 ranked bacterial genera based on relative abundance. The results revealed significant negative correlations between *Rikenellaceae RC9 gut group* and propionate, butyrate, and total VFA. Additionally, *Prevotellaceae UCG-003* exhibited negative correlations with propionate and TVFA, whereas *Ruminococcus* showed a positive correlation with propionate, and, total VFA. The correlation between the isovalerate and *Ruminococcus*, Muribaculaceae_norank, and F082_norank had a significantly positive correlation. *UCG-002* is positively correlated with propionate and negatively correlated with A/P (Figure 4A). These findings suggest a potential role of rumen microorganisms in regulating rumen fermentation.

Correlation network analyses were further performed to investigate the relationships between rumen microorganisms and sheep growth performance. A correlation was defined as having a strong interactive relationship if it had a *p*-value *<*0.05 and an R value >0.4 (Figure 4B). The results suggest that *Veillonellaceae UCG-001*, *Bacteroidales RF16 group_norank*, *Anarovibrio*, and *Gastroanaerophilales_norank* have the potential to influence DMI. Additionally, *Prevotellaceae UCG-001* and *Clostridia UCG-014_norank* are correlated with FBW. While *Lachnospiraceae Unclassified* and *Veillonellaceae UCG-001* has a strong correlation with ADG.

## 4 Discussion

### 4.1 Growth performance

Enzymes secreted by microorganisms during the fermentation process can degrade complex compounds in plant fibers, thereby imparting a distinctive aroma to fermented feed and subsequently enhancing animal intake (24). Research found that feeding fermented rice straw containing probiotics to fattening cattle improved growth performance compared to the Control (25). Similarly, fermented TMR was found to notably increase the feed intake and growth performance of Hanwoo steers (26). However, the findings of the current experiment revealed no significant differences in feed intake among the three groups of Hu sheep, suggesting that the fermented feed did not enhance animal feed intake. This outcome may be attributed to variations in the fermentation strains utilized in different studies, leading to negligible changes in the flavor compounds of the fermented rice husk powder.

Research indicates that the incorporation of fermented feed can positively influence animal growth. For instance, the study conducted in the same area (27) demonstrated that supplementing with 10% fermented mulberry leaves significantly improved the average daily gain of Hu sheep. Furthermore, the fermentation of rice straw using a combination of calcium oxide, *Bacillus* spp., and coconut water markedly increased the digestibility of OM, CP, and DM (28). Additionally, the inoculation of corn silage with either *Lactobacillus buchneri* or *Lactobacillus plantarum* separately improved the intake of dry matter (DM), crude protein (CP), and neutral detergent fiber (NDF) in lambs by Basso et al. (29). In this experiment, the ADG and FBW of the FHR group were significantly higher than those of the other two groups. This finding indicates that fermented rice husk powder increased the ADG and final weight of the sheep without a corresponding increase in feed intake.

### 4.2 Apparent digestibility of nutrients

It is widely acknowledged that microbial fermentation can improve the quality of substandard feed materials (30). Research has demonstrated that treating rice husks through biodegradation by *Pleurotus ostreatus* results in an increase in CP content from 2.15 to 9.3%, alongside a 14.76% rise in digestibility. Additionally, there is a notable reduction in crude fiber and lignin content by 35.4 and 40.9%, respectively (31). The enhancement of nutrient digestibility in animals fed fermented feed is likely attributable to modifications in the physical properties of fibers induced by the fermentation process, which facilitate microbial breakdown in the rumen (32).

The experiment shows that the FHR group’s digestibility is higher than the RH group but lower than the CON group. This implies that fermenting rice husk powder improves its digestibility, though not to the level of the CON group. The difference may be due to the higher lignocellulose content in rice husk compared to soybean stalk powder. The digestibility of CP and EE in fermented rice husk powder was similar to the CON group but significantly higher than the RH group. Fiber digestibility varied among the three groups, suggesting that rice husk powder’s lignocellulose is harder to degrade than soybean stalk powder. However, probiotic fermentation significantly improved its digestibility. Similar results were observed in a study where feeding fermented cottonseed hulls to calves significantly improved their ADG, even though the DM digestibility of the fermented cottonseed hulls was markedly lower than that of the CON group (33). These findings suggest that fermented feeds with high lignocellulose content can enhance digestibility to some extent and promote growth in animals.

### 4.3 Serum biochemical indices

The results of the serum biochemical indicators revealed that the ALB and ALT levels were significantly decreased in the RH and FHR group compared to the CON group, while no significant differences were observed for other indicators, such as GLB. Previous research indicates that increased protein metabolism can result in elevated ALT levels. However, disruptions in hepatocytes cellular membrane may also contribute to higher serum ALT levels (34). Variations in ALB levels partially reflect the nutritional quality of dietary protein and the status of protein digestion, absorption, and metabolism in the animal (35). It is possible that the higher protein content in soybean stalk powder compared to rice husk powder led to the increased ALT and ALB levels in the serum of the Hu sheep in the control group.

### 4.4 Rumen fermentation parameters

Feeding fermented feeds led to a decrease in rumen pH, likely due to microbial facilitation of carbohydrate breakdown during the fermentation process, which subsequently increases VFA production (36). The fermentation process modified the fiber composition of rice husk powder, thereby influencing the rumen fermentation patterns. Notably, the butyrate level in the rumen of the FHR group was significantly elevated compared to the other two groups, while the valerate level was significantly higher than in the RH group. Numerous studies have demonstrated that butyrate plays a crucial role in the development of the rumen epithelium in lambs (37). The marked increase in butyrate levels in the rumen of sheep fed fermented rice husk powder suggests that this dietary intervention may promote rumen development. Furthermore, the A/P in the rumen fluid of the FHR group was significantly lower compared to the control group, indicating that the inclusion of fermented rice husk powder in the diet altered the rumen fermentation pattern.

### 4.5 Rumen microbiota

Rumen microorganisms play a crucial role in fermentation process and overall health. The present study’s diversity analysis results reveal significant alterations in rumen microbial populations due to the different dietary formulations. Previous research indicates that fermented feeds generally do not lead to dramatic shifts in dominant microbial phyla (38). However, in this study, the relative abundances of Acidobacteriota and Chloroflexi were significantly reduced in the FHR group compared to the RH group. At present, the functional roles of Acidobacteriota and Chloroflexi within the rumen remain inadequately understood. It has been observed that feeding a blend of cinnamaldehyde, eugenol, and capsicum oleoresin significantly reduced the relative abundance of Acidobacteriota and Chloroflexi in the rumen, suggesting potential associations with decreased inflammation and apoptosis in the rumen (39).

The FHR group reduced the relative abundances of the Rikenellaceae RC9 gut group, *UCG-010_norank*, *p-251-o5_norank*, *UCG-005*, *Quinella*, and *Lachnospiraceae AC2044 group*. The *Rikenellaceae RC9 gut group* is commonly found in the digestive tracts of various animals and is closely associated with the digestion of carbohydrates and celluloses (40). Additionally, it plays a role in starch degradation (41). Correlation analysis revealed a negative correlation between the *Rikenellaceae RC9 gut group* and various VFA, with a positive correlation observed solely with the A/P. This finding diverges from prior studies, possibly due to differences in the fiber composition of soybean straw powder and fermented rice husk powder. Such differences may result in shifts in the dominant microorganisms involved in degradation and VFA production.

*Ruminococcus* and *Quinella* are primarily associated with the degradation of cellulose and carbohydrates and can produce succinate, a precursor of propionate (42). A strong positive correlation was found between *Ruminococcus* and propionate concentration, suggesting that the dominant microorganisms involved in carbohydrate degradation in the FHR group may be influenced by *Ruminococcus*. The correlation network indicated that Veillonellaceae UCG-001, Bacteroidales *RF16 group_norank*, *Anaerovibrio*, and *Gastranaerophilales_norank* have the potential to regulate DMI in the rumen. *Anaerovibrio* has been reported to generate propionate through lipid metabolism, suggesting its potential role in modulating DMI in lambs (43). Furthermore, the Veillonellaceae family is linked to the remaining feed intake in beef cattle, indicating that *Veillonellaceae UCG-001* may collectively regulate DMI in sheep. The functional ecological niches of different microbial communities present in the rumen interact synergistically, affecting rumen microbial function. Feeding fermented rice husk powder may enrich the microbial network that promotes DMI in sheep, thereby enhancing their growth and development.

## 5 Conclusion

The incorporation of 15% fermented rice husk powder feed has been demonstrated to enhance the growth performance and nutrient digestion of Hu sheep, as well as to increase the concentrations of propionate, butyrate, and valerate in the rumen. This improvement in rumen fermentation and growth performance in sheep is likely mediated by rumen microorganisms, including *Veillonellaceae UCG-001*, *Bacteroidales RF16 group_norank*, *Anaerovibrio*, and *Gastranaerophilales_norank*. Therefore, fermented rice husk powder feed represents a promising alternative to traditional roughage in the diet to enhance animal performance.

## Data Availability

The raw data supporting the conclusions of this article will be made available by the authors, without undue reservation.
