# Supplementary material for: Effects of fermented rice husk powder on growth performance, rumen fermentation, and rumen microbial communities in fattening Hu sheep
Source: Front Vet Sci. 2024 Nov 27;11:1503172. doi: 10.3389/fvets.2024.1503172 (PMC11631883; doi:10.3389/fvets.2024.1503172)
Supplement: Supplementary file 1 [file Table_1.DOCX]

**Appendix 1.** Changes in pH, nutrient ingredients of rice husk powder feed before and after fermentation

| Items |  | Groups |  | *P*-value |
| --- | --- | --- | --- | --- |
|  | RH | FRH | SEM |  |
| pH | 6.50 | 4.17 | 0.01 | <0.001 |
| CP, %DM | 6.39 | 6.45 | 0.08 | 0.776 |
| ASP, %DM | 7.74 | 16.20 | 1.88 | 0.004 |
| EE, %DM | 2.66 | 4.50 | 0.35 | <0.001 |
| Ash, %DM | 11.53 | 11.61 | 0.01 | 0.642 |
| CF, %DM | 36.56 | 36.07 | 0.01 | 0.633 |
| ADF, %DM | 44.27 | 45.49 | 0.51 | 0.275 |
| NDF, %DM | 63.43 | 63.87 | 0.73 | 0.79 |

RH (15% rice husk powder), FHR (15% fermented rice husk powder).
